# Supplementary material for: Analysis of PPARγ Signaling Activity in Psoriasis
Source: Int J Mol Sci. 2021 Aug 10;22(16):8603. doi: 10.3390/ijms22168603 (PMC8395241; doi:10.3390/ijms22168603)
Supplement: Supplementary file 1 [file ijms-22-08603-s001.zip › Supplemental materials_Analysis of PPARg signaling activity in psoriasis/Pathway models/Models images and html files/PPARG inhibits phenotypes assotiated with psoriasis/210764.html]

cell renewal


# Cell Process cell renewal

|  |  |
| --- | --- |
| URN | urn:agi-cellproc:cell%20renewal |
| Total Entities | 60 |
| Connectivity | 1308 |
| Name | cell renewal |

---

|  |  |
| --- | --- |
| ChildConcepts | stem cell renewal |
|  | cancer stem cell renewal |
|  | B-cell renewal |
|  | endothelial cell renewal |
|  | eosinophil renewal |
|  | dendritic cell renewal |
|  | haematopoietic stem cell renewal |
|  | erythroid precursor cell renewal |
|  | B-1a B-cell renewal |
|  | T-lymphoid precursor cell renewal |
|  | memory T-cell renewal |
|  | immunocompetent cell renewal |
|  | myeloid progenitor cell renewal |
|  | hematopoietic cell renewal |
|  | neutrophil renewal |
|  | lymphocyte renewal |
|  | CD8+ memory T-cell renewal |
|  | mature B-cell renewal |
|  | macrophage renewal |
|  | kidney mesangial cell renewal |
|  | leukocyte renewal |
|  | conventional dendritic cell renewal |
|  | CD4+ T-cell renewal |
|  | myeloid cell renewal |
|  | T-cell renewal |
|  | bone marrow stem cell renewal |
|  | erythroid cell renewal |
|  | mast cell renewal |
|  | memory B-cell renewal |
|  | blood platelet renewal |
|  | B-1 B-cell renewal |
|  | monocyte renewal |
|  | histiocyte renewal |
|  | bone marrow derived mesenchymal stem cell renewal |
|  | Langerhans cell renewal |
|  | megakaryocyte renewal |
|  | myeloid derived suppressor cell renewal |
|  | bone marrow stromal cell renewal |
|  | B-lymphoid precursor cell renewal |
|  | plasmacytoid dendritic cell renewal |
|  | erythroblast renewal |
|  | natural killer cell renewal |
|  | regulatory T-cell renewal |
|  | granulocyte renewal |
|  | fibroblast renewal |
|  | microglia renewal |
|  | perivascular macrophage renewal |
|  | CD4+ memory T-cell renewal |
|  | blast cell renewal |
|  | megakaryocyte progenitor cell renewal |
|  | brain capillary endothelial cell renewal |
|  | effector T-cell renewal |
|  | alveolar macrophage renewal |
|  | adipose macrophage renewal |
|  | myofibroblast renewal |
|  | CD8+ T-cell renewal |
|  | multipotent bone marrow stem cell renewal |
|  | tumor infiltrating macrophage renewal |
|  | peritoneal macrophage renewal |
|  | thymocyte renewal |

---

|  |  |
| --- | --- |
| Pathway | PPARG inhibits phenotypes assotiated with psoriasis |

---

|  |  |
| --- | --- |
| MedScan ID | 4203646 |

---

|  |  |
| --- | --- |
| Alias | cell renewal |

---
